# Traumatic Ventral Cervical Spinal Cord Herniation: A Case Report

**DOI:** 10.7759/cureus.4070

**Published:** 2019-02-13

**Authors:** Nicolas K Khattar, Andrew M Donovan, Brent G Oxford, Shawn W C Adams, Thomas J Altstadt

**Affiliations:** 1 Neurological Surgery, University of Louisville School of Medicine, Louisville, USA

**Keywords:** spinal cord injury, trauma, cervical spinal cord, herniation

## Abstract

Spinal cord herniation (SCH) is an uncommon traumatic event that should be considered in patients with vertebral fractures who develop an unusual constellation of autonomic and motor deficits. Herein, we describe a case of rapidly deteriorating neurological function following cervical spine fracture including sequelae such as bilateral lower-extremity weakness, loss of upper extremity motor function, and priapism. Decompression of the spinal cord allowed for the identification of the unusual herniation of the spinal cord and prevention of any further worsening of the neurological injury. Hyperflexion of the cervical spine upon traumatic impact provided the impetus for vertebral retropulsion and subsequent incarceration of the spinal cord. This phenomenon should be considered in the setting of acute traumatic injury to the cervical spinal cord. Surgical intervention is likely to allow the preservation of the remaining neurological function.

## Introduction

Spinal cord herniation (SCH) is a rare condition, often leading to acute neurological complications. SCH is generally classified as idiopathic, iatrogenic, or post-traumatic. Idiopathic SCH is most commonly reported at thoracic levels through a ventral or ventrolateral dural defect [1-2]. Iatrogenic SCH is often postsurgical and has been documented at all levels of the spinal column [3-4]. Traumatic SCH results in acute transdural herniation of the cord from direct dural damage [3,5-6]. Most cases of traumatic SCH present with delayed insidious neurological deterioration following the traumatic event. In very few cases, patients experience immediate post-traumatic neurological deterioration [7]. In this report, we present a case of rapid-onset post-traumatic cervical anterior SCH caused by a C6 burst fracture.

## Case presentation

An 18-year-old male presented to the emergency department after an unwitnessed dive into the shallow waters of a swimming pool while inebriated where he remained submerged for two minutes. Upon extraction, the patient briefly received chest compressions and was resuscitated. The patient complained of shortness of breath, neck pain, and decreased sensory and motor function in the upper and lower extremities.

In the emergency department, the patient was awake, alert, and fully oriented. His motor exam was 5/5 in the elbow flexors and adductors, 3/5 in elbow extensors, 0/5 in finger flexors and abductors with no motor function in bilateral lower extremities. The patient demonstrated a thoracic sensory level at T7. The patient was areflexic, with no clonus or Babinski signs and the patient exhibited priapism. The patient’s clinical examination was consistent with complete spinal cord injury (SCI). This injury represents a class A injury according to the American Spinal Injury Association (ASIA) impairment scale.

**Figure 1:**
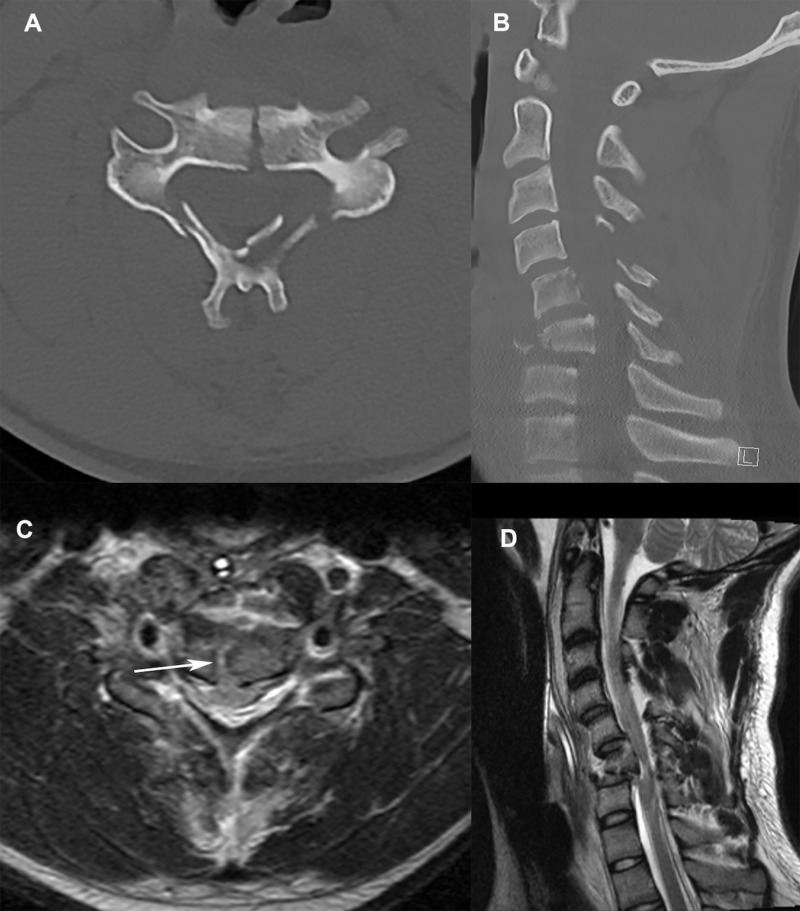
Cervical spine imaging at presentation A. Axial CT of the cervical spine at the C6 level showing the vertebral body fracture as well as the laminar fractures; B. Sagittal CT of the cervical spine shows focal kyphotic deformity at the C6 level associated with the burst fracture with significant canal stenosis; C. Axial MRI of the cervical spine shows herniation of the spinal cord into the vertebral body (arrow); D. Sagittal MRI of the cervical spine showing retropulsion of the C6 vertebral body into the spinal canal with significant spinal cord compression. CT: computed tomography; MRI: magnetic resonance imaging

Computed tomography (CT) of the cervical spine showed a burst fracture of the C6 vertebral body with retropulsion causing severe central canal stenosis with bilateral laminar fractures and inferiorly displaced spinous processes of the C4, C5, and C6 levels (Figures 1A, 1B). Magnetic resonance imaging (MRI) of the cervical spine demonstrated herniation of the spinal cord into the vertebral body of C6 through the burst fracture. This was associated with significant T2 and short T1 inversion recovery (STIR) sequence signal hyperintensity of the central spinal cord from C4 through C7 with foci of gradient echo blooming at C5 and C6 and was indicative of spinal cord contusion and intramedullary hemorrhage (Figures 1C, 1D).

The patient underwent a C6 corpectomy and a C5-C7 anterior interbody arthrodesis with an anterior plate from C5 to C7. Intra-operatively, as the bone fragments were resected, the spinal cord was visualized and a traumatic dural tear was observed with obvious CSF egress. On retrospective review of the MRI, spinal cord herniation into the vertebral body fragment prior to the procedure was noted. The dural defect was closed with a layer of DuraGen (Integra, Plainsboro, NJ) and Tisseel (Baxter, Deerfield, IL). After completion of the anterior procedures, The patient was then placed in the prone position to undergo a C3-T2 posterior lateral fusion followed by C4-C7 laminectomy. No dural lacerations, traumatic lacerations, or CSF was seen posteriorly.

Given the inability to primarily repair the traumatic dural tear, a lumbar drain was used to divert CSF for one week to allow healing of the dural repair. The patient’s hospitalization was complicated by acute respiratory distress syndrome (ARDS) and required multiple bronchoscopies and placement in a Rotoprone bed (Arjo Inc, Addison, IL). The patient subsequently underwent a tracheostomy and feeding tube placement prior to discharge to an acute inpatient rehabilitation facility. The patient completed a six-week course of inpatient rehabilitation and was last seen for follow up at 18 weeks post-injury. At this follow-up, he reported 4+-5/5 strength in bilateral deltoids, 4/5 in bilateral biceps, 3/5 in the right triceps, and 2/5 in the left triceps as well as some regained sensation in the lower extremities. He continues to participate in outpatient physical and occupational therapy.

## Discussion

Herniation of the spinal cord is a rare phenomenon with many different proposed mechanisms including congenital, iatrogenic, idiopathic, and post-traumatic. Herniation from a traumatically acquired dural defect is the least common cause. In the majority of the reported cases of traumatic SCI coming apparently between one and 38 years following a major traumatic event [2,6,8-10]. Symptomatic presentations varied greatly and ranged from unprovoked progressive myelopathic disease to a Brown-Sequard syndrome-like presentation [8-9]. We report a unique case of single-direction cervical SCH from a distinctive traumatic dural defect with the potential for immediate neurologic sequelae.

We hypothesize that the dural tear was likely caused by the retropulsion of fragments of the C6 vertebral body. Neck hyperflexion creates additional space for the spinal cord to herniate between the opposing fragments of the vertebral body causing severe incarceration of the neural elements. The opening and closure of a fracture line have been postulated as a potential mechanism in prior reports [7].

## Conclusions

Traumatic cervical spinal cord herniation is a rare phenomenon that should be considered in patients presenting following major trauma even in the setting of a deteriorating neurological examination. Rapid surgical intervention is then necessary to preserve any possibly salvageable neurological function.
